# Granulomatosis with Polyangiitis: A Focus on Differences and Similarities Between Child and Adult Patients

**DOI:** 10.3390/medicina61030534

**Published:** 2025-03-18

**Authors:** Vincenzo Calabrese, Romina Gallizzi, Alessandra Spagnolo, Mariateresa Zicarelli, Diana Sutera, Alessandra Farina, Valeria Cernaro, Domenico Santoro

**Affiliations:** 1 Department of Medicine and Surgery, University of Enna “Kore”, 94100 Enna, Italy; v.calabrese@outlook.it; 2 Unit of Pediatric Nephrology and Rheumatology, University of Messina, 98125 Messina, Italy; rgallizzi@unicz.it (R.G.); alessandraspagnolo93@gmail.com (A.S.); dianasutera@icloud.com (D.S.); 3 Unit of Nephrology and Dialysis, Department of Clinical and Experimental Medicine, A.O.U. “G. Martino”, University of Messina, 98125 Messina, Italy; mteresa.zicarelli@gmail.com; 4 Department of Medical and Surgical Sciences, University of Catanzaro “Magna Graecia”, 88100 Catanzaro, Italy; alessandrafarina999@gmail.com (A.F.); vcernaro@unime.it (V.C.)

**Keywords:** Wegener’s disease, child, adult, kidney damage

## Abstract

Wegener’s granulomatosis (WG), or granulomatosis with polyangiitis (GPA), is a rare autoimmune disease that can cause inflammation in various organs, including the kidneys. Renal involvement in GPA is a major cause of morbidity and mortality in both adults and children, and early detection and effective treatment are essential for preventing renal failure. This review aims to summarize the current evidence on the incidence, clinical features, treatment, and outcomes of renal involvement in children with Wegener’s granulomatosis. The incidence of renal involvement in children with GPA ranged from 26% to 56%. Renal involvement is a common and serious complication of GPA in children, and early detection and effective treatment are crucial for preventing renal failure. The most common clinical features were proteinuria, hematuria, and reduced glomerular filtration rate. The majority of children with renal involvement in GPA required treatment with corticosteroids and immunosuppressive agents. The treatment outcomes varied among the studies, with some children achieving remission of renal involvement while others developed end-stage renal disease. Although most features are the same in children and adult patients, this review summed up some important differences between these two different populations. Further studies are needed to identify the most effective treatment strategies for renal involvement in children with GPA.

## 1. Introduction

Wegener’s granulomatosis, or granulomatosis with polyangiitis (GPA), is predominantly a small vessel vasculitis associated with antineutrophil cytoplasmic antibodies (ANCA) with clinical picture preference for the involvement of the upper airways, lungs and kidneys. It occurs at all ages, and is one of the most common vasculitides, with incidence rates between 0.03 and 3.2 × 100,000 children per year [1]. There is a north–south gradient, with a higher frequency in Nordic countries. GPA affects both sexes. While the mean age of occurrence is 45 years, some cases have been described in older adults and in children.

The etiology is unknown, but ANCA, antineutrophil antibodies in circulation, directed against proteinase-3 (PR3), predispose to autoimmune pathogenesis.

Upon recruitment to sites of inflammation, these neutrophils play a crucial role in microbial defense but may also contribute to tissue damage. The PR3 autoantigen, displayed on the surface of apoptotic neutrophils, interferes with their clearance by macrophages. Furthermore, PR3 expression on the membranes of activated neutrophils hinders the resolution of inflammation and serves as a key contributor to the pathogenesis of GPA. While the physiological phagocytosis of apoptotic cells generally exerts anti-inflammatory effects, PR3 is recognized as a danger signal by macrophages, which, in turn, trigger an immune response. This maladaptive action of PR3 plays a significant role in the initiation of autoimmune processes, particularly by activating plasmacytoid dendritic cells. These dendritic cells, in response, cease production of CD4+ regulatory T cells and instead facilitate the differentiation of helper T cells with a Th9/Th2 cytokine profile.

However, the presence of concomitant factors such as infections and environmental factors also appears to be needed for the disease to manifest. Although many aspects are common in adults and children, some characteristics are differently manifested based on age. Indeed, GPA in childhood is often complicated by subglottic stenosis and nasal deformity, whereas treatment-related morbidity and malignancies are less common than in adults. The introduction of combined treatment with cyclophosphamide and glucocorticoids led to a considerable improvement in clinical results; however, relapses of the disease and the risk of chronic organ damage at all ages need a long-term follow-up in all patients. New therapeutic regimens are needed to avoid these relapses [2]. It is a potentially life-threatening condition that can affect various organs, including the kidneys. The involvement of the renal system in children with Wegener’s disease has been a subject of interest in recent years, but the impact on pediatric patients has not been thoroughly studied. This review aims to provide a comprehensive overview of the current evidence on the clinical manifestations, diagnosis, and treatment of Wegener’s disease with renal involvement in children. Furthermore, this review aims to provide insight into the management of pediatric patients with this condition and help guide future research in this field and summarize the major differences in the GPA manifestation in adults and children. Indeed, highlighting the major features and the major differences helps different clinicians to better diagnose the GPA in different patient groups [3].

## 2. Wegener’s Granulomatosis in Children

GPA in children is rare, causing a lack of evidence in the literature. Indeed, the reports described in the literature are few, often presented as case reports, case series, and literature reviews. These studies confirmed that GPA in children has many features in common with adult disease, mostly for lung and kidney involvement. However, some differences exist between them, including a predominance in female patients and a higher incidence of subglottic stenosis in the pediatric population.

Other aspects of the disease in children are incompletely defined, such as the prevalence of ANCA, which has been rarely reported in the pediatric working group despite its diagnosis criteria being increasingly met [4,5]. According to the EULAR/PReS endorsed consensus criteria, three of the following six features should be present: abnormal urinalysis (hematuria and/or significant proteinuria); granulomatous inflammation on biopsy; nasal sinus inflammation; subglottic, tracheal, or endobronchial stenosis; abnormal chest X-ray; or CT, PR3 ANCA or C-ANCA staining. The use of specific criteria is fundamental to provide a common research language and to identify and better classify the diseases based on the specific features that can vary across different age strata. Furthermore, the ANCA detection should be considered for each GPA suspect, in keeping with the cited criteria.

If a kidney biopsy is performed, it characteristically shows necrotizing pauci-immune glomerulonephritis. Furthermore, kidney biopsy is the gold standard for the diagnosis, and it is able to detect other forms of rapidly progressive glomerulonephritis.

J.D. Akikusa et al. [6], in a study of 25 pediatric GPA patients retrieved in a single center, showed a significant female predominance with a male/female ratio of 1:4, and these data are in accord with two large pediatric studies. However, it is a debated subject due to non-unique results in the literature [6,7]. The age of onset of symptoms is generally the adolescent period; again the data are consistent with that of several studies [4,8,9], although in contrast with Westman et al. [3].

In the study by J. D. Akikusa et al., in both adults and children, most of the patients presented generalized GPA at diagnosis with renal involvement. In the reports of Hoffman et al. [7] and Rottem et al. [8], 18% of adults and 9% of children had renal disease at presentation, most of them asymptomatic. In comparison, at presentation, 88% of patients in the J.D. Akikusa et al.’s study had renal involvement, nearly 30% had elevated serum creatinine, and 20% required dialysis. All six patients under the age of 18 in the study of Stegmayr et al. [10] and all four in the report by Hall et al. [11] had renal involvement at presentation. Loss of renal function in patients in the study by J.D. Akikusa et al. was common—at the last follow-up, 40% had a sustained increase in serum creatinine and 12% had end-stage renal disease, figures comparable with those in the study by Rottem et al. (35% and 9%, respectively) [6,8]. This finding was rarely reversible. Indeed, only 50% of patients who required dialysis during the course of their illness regained sufficient renal function to permit discontinuation of dialysis in J.D. Akikusa’s analysis [6]. It has been suggested that plasmapheresis is potentially useful in patients with ANCA-associated vasculitis requiring dialysis acutely [11]. Pulmonary disease is a common presenting feature, with pulmonary hemorrhage often occurring as part of a pulmonary-renal syndrome, and is as common as nodular lesions at diagnosis.

Lung involvement was also common in disease exacerbations, even though pulmonary hemorrhage was less frequent. Indeed, previous investigators found that only 34–41% of patients with pulmonary radiographic changes had pulmonary symptoms [4,6].

Similar airway congestion is found in children and adults [4,6], even though the incidence of subglottic stricture seems to be higher in the pediatric population than in the adult work group [4,12].

**Figure 1:**
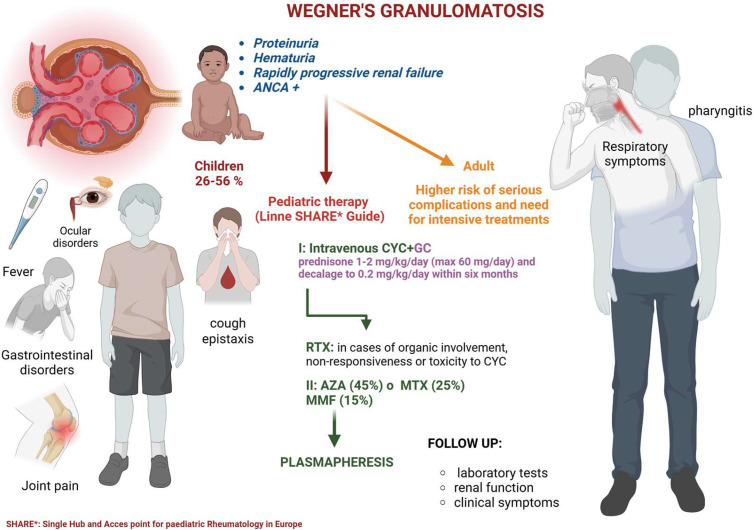
Symptoms and treatment of Wegener’s disease. AZA: azathioprine; CYC: cyclophosphamide; GC: glucocorticoids; MMF: mycophenolate; MTX: metrotexate.

The renal involvement in Wegener’s disease is a major concern as it can lead to serious complications, such as glomerulonephritis, renal failure, and death in more severe cases. In children, the diagnosis of Wegener’s disease can be challenging, and there is a need for a better understanding of its clinical presentation, diagnostic criteria, and treatment options in this population. To better understand the impact of Wegener’s disease on pediatric patients, this review will examine the available evidence from peer-reviewed journals and other relevant sources. Clinically, children with Wegener’s disease may present with symptoms such as fever, weight loss, respiratory symptoms (such as coughing, shortness of breath, or bloody sputum), joint pain, and eye symptoms (such as redness, pain, or vision changes). The diagnosis of Wegener’s disease in children can be confirmed through a combination of clinical evaluation, laboratory tests, and imaging studies. Antineutrophil cytoplasmic antibody (ANCA) testing is a commonly used laboratory test to diagnose Wegener’s disease. However, ANCA testing is not always positive in children with this condition, and a negative test result does not exclude the diagnosis. Renal involvement in Wegener’s disease is a major concern as it can lead to serious complications, such as glomerulonephritis, renal failure, and in severe cases, death. In children with Wegener’s disease, renal involvement can present as proteinuria, hematuria, or rapidly progressive renal failure. The diagnosis of renal involvement in Wegener’s disease in children can be challenging. Healthcare professionals need to consider this condition in children presenting with unexplained renal symptoms [13] (Figure 1).

## 3. Outcome and Treatment in Adults and Children

The outcome of Wegener’s disease in children depends on several factors, including the severity of the disease, the presence of renal involvement, and the promptness and effectiveness of treatment.

The prognosis of GPA is assessed using the five-factor score (FFS), with the updated version applicable to GPA. This scoring system evaluates five clinical features, each contributing one point, that are associated with an increased risk of mortality: age > 65 years, presence of specific cardiomyopathy, gastrointestinal involvement, renal impairment characterized by a stabilized serum creatinine level >150 μmol/L, and the absence of ENT (ear, nose, and throat) manifestations. While the FFS provides valuable insight into the prognosis of GPA, it does not influence treatment decisions, similarly to its application in other forms of necrotizing vasculitis.

Children with Wegener’s disease who receive early and appropriate treatment have a better chance of remission and avoiding serious complications, such as renal failure [14].

In general, the treatment of Wegener’s disease in children involves a combination of immunosuppressive medications, such as cyclophosphamide or azathioprine, and corticosteroids. The use of immunosuppressive medications is aimed at suppressing the immune system and reducing inflammation in affected organs. Corticosteroids are used to reduce inflammation and swelling, and they can be tapered once the disease is under control [13].

In severe cases of Wegener’s disease in children, other treatments may be necessary, such as rituximab, a monoclonal antibody, or plasmapheresis, a procedure that involves removing harmful antibodies from the blood. The choice of treatment will depend on the severity of the disease and the presence of renal involvement.

As highlighted, the lack of RCT did not allow us to obtain sufficient evidence about the new treatment. Indeed, for Avacopan only a few case reports have been published. Although they reported complete remission, they are not enough to guarantee the safety and efficacy of this therapy [15,16]. In terms of outcome measures, the goal of treatment for Wegener’s disease in children is to achieve remission, prevent serious complications, and improve quality of life. Outcome measures in children with Wegener’s disease typically include laboratory tests (such as ANCA testing), kidney function tests, and assessments of clinical symptoms (such as respiratory symptoms and joint pain).

In conclusion, the outcome and treatment of Wegener’s disease in children is a complex and multidisciplinary process that requires a team approach involving pediatric specialists, rheumatologists, and nephrologists. The prompt and effective treatment of Wegener’s disease in children is crucial for preventing serious complications and improving patient outcomes.

The outcome and treatment of Wegener’s disease in adults are similar to those in children, but some differences need to be focused on.

The goal of treatment for Wegener’s disease in both children and adults is to achieve remission and prevent serious complications, such as renal failure. However, the use of immunosuppressive medications in adults may be more complex, as they may have more co-existing medical conditions or a longer duration of disease.

In terms of treatment, adult patients with Wegener’s disease may require higher doses of immunosuppressive medications, such as cyclophosphamide or azathioprine, to achieve remission. Corticosteroids are also used in adult patients with Wegener’s disease to reduce inflammation and swelling. In severe cases of Wegener’s disease in adults, other treatments may be necessary, such as rituximab or plasmapheresis.

The pediatric-specific SHARE (Single Hub and Access point for paediatric Rheumatology in Europe) guidelines endorse the use of intravenous cyclophosphamide (CYC) in combination with glucocorticoids (GCs) for the treatment of pediatric vasculitis. The use of rituximab (RTX) may be considered in cases of critical organ involvement or life-threatening disease that fail to respond to conventional vasculitis therapies, or in patients with concerns regarding CYC-related toxicity. The remission maintenance strategies recommended in the SHARE guidelines for first-line therapy, involving azathioprine (AZA), methotrexate (MTX), mycophenolate mofetil (MMF), or RTX in conjunction with low-dose GCs, are broadly aligned with adult-based therapeutic recommendations.

The two intravenous CYC regimens (pediatric adaptations of the European Vasculitis Study Group [EUVAS] and National Institutes of Health [NIH] adult protocols) are typically administered for a minimum of 3 months, continuing until sustained disease inactivity is achieved, and for a maximum duration of 6 months.

Among pediatric rheumatologists surveyed, a modified two-dose regimen of RTX (750 mg/m^2^) or a four-dose regimen (375 mg/m^2^) was regarded as therapeutically equivalent. For maintenance therapy, 70% of respondents utilized either AZA (45%) or MTX (25%), while fewer than 15% employed MMF, which appears to be less effective than AZA. Notably, none of the respondents included leflunomide as a maintenance option. MMF was considered a viable maintenance therapy by only 31.8% (7/22) of the surveyed practitioners.

AZA and MTX are recommended for remission maintenance, even though rituximab can be considered a new recommendation [17].

Despite well-documented toxicity concerns, high-dose glucocorticoids remain a fundamental component of remission induction in pediatric patients. The use of GCs in pediatric antineutrophil cytoplasmic antibody (ANCA)-associated vasculitis exhibits considerable variability, and the potential for dose-related toxicities, particularly those affecting growth and self-image, must be carefully considered. The recommended initial dose is prednisone at 1–2 mg/kg/day (maximum 60 mg/day), with a tapering to 0.2 mg/kg/day (or 10 mg/day, whichever is lower) by the sixth month of treatment.

Although the efficacy of plasmapheresis as an adjunctive treatment for adult patients has been historically limited and controversial, reports have described its use in pediatric ANCA-associated vasculitis. Among the surveyed pediatric rheumatologists, 75% recommended plasmapheresis for cases of rapidly progressive renal disease or severe pulmonary hemorrhage.

In terms of outcome measures, laboratory tests (such as ANCA testing), kidney function tests, and assessments of clinical symptoms (such as respiratory symptoms and joint pain) are important indicators of the effectiveness of treatment in both adult and pediatric patients. However, the longer duration of the disease in adult patients may increase the risk of serious complications and a less favorable outcome.

In conclusion, the outcome and treatment of Wegener’s disease in adults and children are similar, but adult patients may require more intensive treatment and have a higher risk of serious complications. The management of Wegener’s disease in adults requires a multidisciplinary approach involving rheumatologists, nephrologists, and other specialists to ensure the best possible outcome [9,13,18,19,20].

## 4. Wegener and Kidney

Similarly to adult patients, childhood GPA includes necrotizing granulomatosis of the respiratory tract including nose and throat in about 80% of the cases, necrotizing vasculitis, renal involvement as glomerulonephritis in about 70% of the patients (GN). Typical symptoms of childhood GPA consist of fever, anorexia, and weight loss [6]. Although less frequently involved, symptoms also include arthralgia and myalgia (64%), gastrointestinal symptoms (42%), eye symptoms (37%), skin symptoms (35%), arthritis (32%), and nervous system symptoms (25%) [1,21]. Renal manifestations vary from mild urinary sediment abnormalities or renal dysfunction to acute kidney damage requiring dialysis where oliguria was related to poorer outcomes [22]. In detail, Ayala de la Cruz Mdel C. [23] reported a case of a 12-year-old female presenting with high blood pressure, macroscopic hematuria, and edema as early symptoms of GPA. Furthermore, Ziółkowska H. et al. [24] reported a case of pediatric ERSD on a total of 195 pediatric dialyses. Childhood renal biopsy reveals segmental necrotizing glomerulonephritis in almost the totality of the issues, whereas vasculitis occurs in about half of the biopsies [25]. Nevertheless, renal biopsy was not able to help in the diagnosis of the early stage of GPA [26]. Furthermore, overlap syndrome with LES was reported. In the details, Oner et al. [27] reported a case of initial SLE diagnosis with lupus glomerulonephritis class IV, followed by negativization of ANA after steroid and cyclophosphamide infusion, and subsequent diagnosis of GPA overlap after nasal septum perforation. Furthermore, Chron’s disease and sarcoidosis could overlap with GPA, according to Makovetskaya G. et al. [28].

Albeit infrequent, electrolyte disturbances were also found in Wegener’s disease. In detail, X. Bosch et al., as well as Endelson G.W. et al. and Shaker et al. [29,30,31], reported a few cases of hypercalcemia and hyperparathyroidism in patients with excess vitamin D, with complete resolution of this manifestation after steroid therapy.

The majority of children with renal involvement in Wegener’s granulomatosis required treatment with corticosteroids and immunosuppressive agents, with some studies reporting positive outcomes such as remission of proteinuria and improvement in renal function.

Several of the included studies also reported on the use of alternative treatment options, such as plasma exchange and rituximab, with varying levels of success. The use of these treatments was often limited by the small sample size and heterogeneity of the patient populations, making it difficult to draw firm conclusions about their efficacy in treating renal involvement in children with Wegener’s granulomatosis.

Renal replacement treatments, from dialysis or transplantation, should be considered in case of renal failure.

## 5. Renal Outcome in Childhood GPA

Although prevalence differs in various studies, CKD and ESRD could be a consequence of Wegener’s disease. For example, Stegmayr B.G. et al. [10] reported that in a total of ten patients, one case of severe kidney relapse featured massive proteinuria and ESRD, followed by loss of the graft five years after transplantation. Similarly, in a case series reported by Siomou et al. [22], one patient out of a total of seven children affected by GPA, developed ERSD, and only three patients maintained a normal renal function in a long-term follow-up. Furthermore, as reported by Michael Frosch et al. and by Valentini R.P. et al. [2,32], pediatric CKD was the major point of long-term mortality and ERSD. Indeed, Valentini’s work group reported three ESRD in a follow-up of 48 ± 12 months on seven enrolled patients.

A higher rate of ESRD was reported by Aasarød K. et al. [33], who reported 23% of ESRD with about 75% renal survival at 5 years in a retrospective analysis of 108 patients. According to Belostosky V.M. et al. [4], patients who manifested GPA before the age of 5 years seem to have fewer kidney manifestations than patients who had ESRD, based on a follow-up of those who manifested symptoms after the age of 5 years. Conversely, mortality was not related either to onset age or kidney involvement [34], but it was not specified if transplanted patients were excluded from the analysis, and stratification for the race was not performed, although Hogan S.L. reported that African Americans had a worse outcome than Caucasian children [35]. However, in the same study, Hogan’s analysis confirmed the absent impact of kidney diseases on mortality. However, the observational studies conducted on dialysis-dependent children showed a higher mortality rate, as reported by Mekhail T.M. et al. [36] who showed a mortality rate of about 50%.

Wegener’s disease did not seem to have several renal manifestations among the ANCA-related vasculitis. Indeed, Meng T. et al. [37] in a case series of Chinese pediatric patients affected by ANCA-related vasculitis, reported 56% of ERSD in a follow-up of 46.3 months (standard deviation of 36 months), higher than the rates previously cited studies on GPA, and Cabreal D.A. et al. [38] reported that MPA had more several kidney manifestations compared to GPA despite the similar onset age.

The results of the systematic review showed that the incidence of renal involvement in children with Wegener’s granulomatosis ranged from 26% to 56%. The most common clinical features of renal involvement in children with Wegener’s granulomatosis were proteinuria, hematuria, and reduced glomerular filtration rate. Additionally, renal biopsy was performed in some cases to confirm the diagnosis of Wegener’s granulomatosis.

The majority of children with renal involvement in Wegener’s granulomatosis required treatment with corticosteroids and immunosuppressive agents. The treatment response was good in the majority of cases, with improvement in clinical and laboratory parameters. However, in some cases, the treatment was not effective, and the disease progressed, leading to end-stage renal disease.

In conclusion, renal involvement is a frequent manifestation of Wegener’s granulomatosis in children, with a wide range of clinical presentations and severity. Effective treatment with corticosteroids and immunosuppressive agents is crucial for achieving a good outcome in children with Wegener’s granulomatosis and renal involvement. However, further studies are needed to determine the optimal treatment strategy and the long-term outcomes of children with Wegener’s granulomatosis and renal involvement.

## 6. Difference Between Children and Adults

Wegener’s disease can affect both children and adults, but there are some differences between the two groups in terms of symptoms, diagnosis, and treatment. Children tend to have milder symptoms and a better chance of achieving remission with less intensive treatment, while adults have a higher risk of serious complications and may require more intensive treatment.

In detail, children may present with nasal congestion and epistaxis, while adults are more likely to have symptoms such as sinusitis, joint pain, and lung involvement. The diagnosis of Wegener’s disease may be more challenging in children due to the milder symptoms and the lack of specific diagnostic criteria, even though the EULAR/PReS endorsed consensus criteria, which detail three useful criteria with diagnostic and prognostic relevance, were established. Children may also be more likely to experience side effects from immunosuppressive medications, which can affect their growth and development.

Overall, it is important to monitor the outcome of treatment in both children and adults with Wegener’s disease and to tailor treatment to the individual patient. Further research is needed to better understand the best methods for managing and treating this disease in both children and adults.

The goal of treatment for Wegener’s disease is to achieve remission and prevent serious complications, such as renal failure. The outcome and treatment of Wegener’s disease in adults are similar to those in children, but adult patients may require more intensive treatment and have a higher risk of serious complications.

Indeed, although adult patients with Wegener’s disease may require higher doses of immunosuppressive medications, such as cyclophosphamide or azathioprine, rituximab, or plasmapheresis in severe cases., children may have a better chance of achieving remission with less intensive treatment and are typically treated with lower doses of immunosuppressive medications due to concerns about side effects and potential long-term consequences.

## 7. Conclusions

In summary, although Wegener’s disease is not frequent in children, renal involvement is highly prevalent in these patients, including ESRD. Furthermore, it increases the mortality risk by up to 50%. For this reason, early diagnosis would allow us to reduce mortality and several morbidities in this disease if treatment with rtx, cyclophosphamide or methylprednisone was started early [39,40].

In this review, the incidence of renal involvement in children with Wegener’s granulomatosis varied among the studies included, ranging from 26% to 56%. The most common clinical features associated with renal involvement in children with Wegener’s granulomatosis were proteinuria, hematuria, and reduced glomerular filtration rate. Overall, the results of this review suggest that renal involvement in children with Wegener’s granulomatosis is a common and potentially serious complication that requires prompt and aggressive treatment. Further research is needed to better understand the best approach to managing this condition in children, including the use of alternative treatment options and the impact of early treatment on long-term outcomes.

Further well-structured studies, RCTs for treatment or observational studies to identify risk factors or features related, should be conducted, with a large number of centers involved to ensure a valid sample size.

This review focused on the different organs involved, highlighting the importance of comprehensive knowledge of their features and the differences based on the patient’s age. Indeed, the management of Wegener’s disease in adults requires a multidisciplinary approach involving rheumatologists, nephrologists, and other specialists to ensure the best possible outcome. In conclusion, it is important to monitor the outcome of treatment in both children and adults with Wegener’s disease, using laboratory tests, kidney function tests, and assessments of clinical symptoms. Further research is needed to better understand the best methods for managing and treating this disease in both children and adults.

## References

[1] Cabral D.A., Uribe A.G., Benseler S., O’Neil K.M., Hashkes P.J., Higgins G., Zeft A.S., Lovell D.J., Kingsbury D.J., Stevens A. (2009). Classification, presentation, and initial treatment of Wegener’s granulomatosis in childhood. Arthritis Rheum..

[2] Frosch M., Foell D. (2004). Wegener granulomatosis in childhood and adolescence. Eur. J. Pediatr..

[3] Westman K.W., Stone J.H. (2015). Wegener granulomatosis. Rheum. Dis. Clin. N. Am..

[4] Belostotsky V.M., Shah V., Dillon M.J. (2002). Clinical features in 17 paediatric patients with Wegener granulomatosis. Pediatr. Nephrol..

[5] Bartůňková J., Tesař V., Šedivá A. (2003). Diagnostic and pathogenetic role of antineutrophil cytoplasmic autoantibodies [review]. Clin. Immunol..

[6] Akikusa J.D., Schneider R., Harvey E.A., Hebert D., Thorner P.S., Laxer R.M., Silverman E.D. (2007). Clinical features and outcome of pediatric Wegener’s granulomatosis. Arthritis Rheum..

[7] Hoffman G.S., Kerr G.S., Leavitt R.Y., Hallahan C.W., Lebovics R.S., Travis W.D., Rottem M., Fauci A.S. (1992). Wegener granulomatosis: An analysis of 158 patients. Ann. Intern. Med..

[8] Rottem M., Fauci A.S., Hallahan C.W., Kerr G.S., Lebovics R., Leavitt R.Y., Hoffman G.S. (1993). Wegener granulomatosis in children and Pediatric Wegener’s Granulomatosis 843 adolescents: Clinical presentation and outcome. J. Pediatr..

[9] Halstead L.A., Karmody C.S., Wolff S.M. (1986). Presentation of Wegener’s granulomatosis in young patients. Otolaryngol. Head Neck Surg..

[10] Stegmayr B.G., Gothefors L., Malmer B., Müller D.E., Nilsson K., Sundelin B. (2000). Wegener granulomatosis in children and young adults: A case study of ten patients. Pediatr. Nephrol..

[11] Hall S.L., Miller L.C., Duggan E., Mauer S.M., Beatty E.C., Hellerstein S. (1985). Wegener granulomatosis in pediatric patients. J. Pediatr..

[12] Gaskin G., Pusey C.D. (2001). Plasmapheresis in antineutrophil cytoplasmic antibody-associated systemic vasculitis. Ther. Apher..

[13] Stone J.H., Merkel P.A., Spiera R., Seo P., Langford C.A., Hoffman G.S., Kallenberg C.G., Clair E.W.S., Turkiewicz A., Tchao N.K. (2010). Rituximab versus cyclophosphamide for ANCA-associated vasculitis. N. Engl. J. Med..

[14] Potentas-Policewicz M., Fijolek J. (2024). Granulomatosis with polyangiitis: Clinical characteristics and updates in diagnosis. Front. Med..

[15] Bober E., Sharma B., Papasavvas I., McAdoo S., Petrushkin H. (2025). Avacopan in the treatment of refractory scleritis secondary to granulomatosis with polyangiitis: A case report. Eur. J. Ophthalmol..

[16] Ennis D., Yeung R.S., Pagnoux C. (2020). Long-term use and remission of granulomatosis with polyangiitis with the oral C5a receptor inhibitor avacopan. BMJ Case Rep..

[17] Morishita K.A., Wagner-Weiner L., Yen E.Y., Sivaraman V., James K.E., Gerstbacher D., Szymanski A.M., O’Neil K.M., Cabral D.A., Childhood Arthritis and Rheumatology Research Alliance (CARRA) Antineutrophil Cytoplasmic Antibody–Associated Vasculitis Workgroup (2022). Consensus Treatment Plans for Severe Pediatric Antineutrophil Cytoplasmic Antibody-Associated Vasculitis. Arthritis Care Res..

[18] Jayne D. (2019). SP0182 treatment of AAV. Ann. Rheum. Dis..

[19] Daikeler T., Kistler A.D., Martin P.Y., Vogt B., Huynh-Do U. (2015). The role of rituximab in the treatment of ANCA-associated vasculitides (AAV). Swiss Med. Wkly..

[20] Miloslavsky E.M., Specks U., Merkel P.A., Seo P., Spiera R., Langford C.A., Hoffman G.S., Kallenberg C.G., St Clair E.W., Tchao N.K. (2014). Rituximab in ANCA-Associated Vasculitis-Immune Tolerance Network Research Group. Rituximab for the treatment of relapses in antineutrophil cytoplasmic antibody-associated vasculitis. Arthritis Rheumatol..

[21] A Plumb L., Oni L., Marks S.D., Tullus K. (2018). Paediatric anti-neutrophil cytoplasmic antibody (ANCA)-associated vasculitis: An update on renal management. Pediatr. Nephrol..

[22] Siomou E., Tramma D., Bowen C., Milford D.V. (2012). ANCA-associated glomerulonephritis/systemic vasculitis in childhood: Clinical features–outcome. Pediatr. Nephrol..

[23] Ayala de la Cruz Mdel C., González Díaz R., López Lara N.D. (2003). Granulomatosis de Wegener. Reporte de un caso pediátrico y revisión de la literatura [Wegener’s granulomatosis. Report of a pediatric case and review of the literature]. Rev. Alerg. Mex..

[24] Ziółkowska H., Adamczuk D., Leszczyńska B., Roszkowska-Blaim M. (2009). Kłebuszkowe choroby nerek jako przyczyna schyłkowej niewydolności nerek [Glomerulopathies as causes of end-stage renal disease in children]. Pol. Merkur. Lekarski..

[25] Lie J.T. (1990). Members and Consultants of the American College of Rheumatology Subcommittee on Classification of Vasculitis. Illustrated histopathologic classification criteria for selected vasculitis syndromes. Arthritis Rheum..

[26] Aasarød K., Bostad L., Hammerstrøm J., Jørstad S., Iversen B.M. (2001). Renal histopathology and clinical course in 94 patients with Wegener’s granulomatosis. Nephrol. Dial. Transpl..

[27] Oner A., Memis L., Kiper N., Erdoğan Ö., Demircin G., Bülbül M., Üner Ç. (2004). A boy with consecutive development of SLE and Wegener granulomatosis. Pediatr. Nephrol..

[28] Makovetskaya G., Mazur L., Balashova E. (2019). Granulomatous Interstitial Nephritis in Children Resulting from Wegener’s Granulomatosis, Crohn’s Disease, or Sarcoidosis. Sarcoidosis and Granulomatosis-Diagnosis and Management.

[29] Bosch X., Lopez-Soto A., Morello A., Olmo A., Urbano-Marqlez A. (1997). Vitamin D metabolite-mediated hypercalcemia in Wegener’s granulomatosis. Mayo Clin. Proc..

[30] Edelson G.W., Talpos G.B., Bone H.G. (1993). Hypercalcemia associated with Wegener’s granulomatosis and hyperparathyroidism: Etiology and management. Am. J. Nephrol..

[31] Shaker J.L., Redlin K.C., Warren G.V., Findling J.W. (1994). Case report: Hypercalcemia with inappropriate 1,25-dihydroxyvitamin D in Wegener’s granulomatosis. Am. J. Med. Sci..

[32] Valentini R.P., Smøyer W.E., Sedman A.B., Kershaw D.B., Gregory M.J., Bunchman T.E. (1998). Outcome of antineutrophil cytoplasmic autoantibodies-positive glomerulonephritis and vasculitis in children: A single-center experience. J. Pediatr..

[33] Aasarød K., Iversen B.M., Hammerstrøm J., Bostad L., Vatten L., Jørstad S. (2000). Wegener’s granulomatosis: Clinical course in 108 patients with renal involvement. Nephrol. Dial. Transplant..

[34] Bajema I.M., Hagen E., Van Der Woude F.J., Bruijn J.A. (1997). Wegener’s granulomatosis: A meta-analysis of 349 literary case reports. J. Lab. Clin. Med..

[35] Hogan S.L., Nachman P.H., Wilkman A.S., Jennette J.C., Falk R.J. (1996). Prognostic markers in patients with antineutrophil cytoplasmic autoantibody-associated microscopic polyangiitis and glomerulonephritis. J. Am. Soc. Nephrol..

[36] Mekhail T.M., Hoffman G.S. (2000). Longterm outcome of Wegener’s granulomatosis in patients with renal disease requiring dialysis. J. Rheumatol..

[37] Meng T., Shen C., Tang R., Lin W., Ooi J.D., Eggenhuizen P.J., Zhou Y.-O., Chen J., He F., Xiao Z. (2022). Clinical features and outcomes of anti-neutrophil cytoplasmic autoantibody-associated vasculitis in Chinese childhood-onset patients. Clin. Exp. Med..

[38] Cabral D.A., Canter D.L., Muscal E., Nanda K., Wahezi D.M., Spalding S.J., Twilt M., Benseler S.M., Campillo S., Charuvanij S. (2016). Comparing Presenting Clinical Features in 48 Children With Microscopic Polyangiitis to 183 Children Who Have Granulomatosis With Polyangiitis (Wegener’s): An ARChiVe Cohort Study. Arthritis Rheumatol..

[39] Stein S., Miller L., Konnikov N. (1998). Wegener’s granulomatosis: Case report and literature review. Pediatr. Dermatol..

[40] Lee P.Y., Adil E.A., Irace A.L., Neff L., Son M.B.F., Lee E.Y., Perez-Atayde A., Rahbar R. (2017). The presentation and management of granulomatosis with polyangiitis (Wegener’s Granulomatosis) in the pediatric airway. Laryngoscope.

